# Analysis of the Gut Microflora in Patients With Parkinson's Disease

**DOI:** 10.3389/fnins.2019.01184

**Published:** 2019-11-22

**Authors:** Miao Jin, Jing Li, Fei Liu, Na Lyu, Kang Wang, Lu Wang, Shihao Liang, Hua Tao, Baoli Zhu, Rashad Alkasir

**Affiliations:** ^1^Neurology Department of China - Japan Friendship Hospital, Beijing, China; ^2^Institute of Microbiology, Chinese Academy of Sciences, Beijing, China; ^3^Savaid Medical School, University of Chinese Academy of Sciences, Beijing, China; ^4^Collaborative Innovation Center for Diagnosis and Treatment of Infectious Diseases, The First Affiliated Hospital, College of Medicine, Zhejiang University, Hangzhou, China

**Keywords:** Parkinson's disease, gut microflora, Illumina MiSeq, 16S rRNA gene, *Prevotella*, *Turicibacter*

## Abstract

This study was conducted to explore the composition of the fecal microflora of Chinese Parkinson's disease (PD) patients, as well as to explore links between PD clinical features and antiparkinsonian medications on the gut microflora. Seventy-two PD cases [59 patients suffering from PD for >1 year (OPD) and 13 new PD (NPD) patients] were studied. Microflora communities in the feces of the patients and corresponding healthy controls (HCs) were examined using high-throughput Illumina MiSeq sequencing targeting the 16S rRNA gene. The gut microflora of OPD patients contained high levels of Rikenellaceae compared to corresponding HCs. In addition, significantly higher levels of Turicibacteraceae were found in the NPD group compared to the corresponding HCs. The genera *Turicibacter* and *Prevotella* were significantly correlated with the PD severity scores. Our findings that some fecal microflora were closely related to PD clinical characteristics may enhance our understanding of the pathogenesis and treatment of PD.

## Introduction

Microflora is now considered as an important part of our body. Indeed, the human body is considered to harbor 10-fold more microbial cells than human cells, and these flora carry nearly 100 to 200 times more protein-coding genes than the human genome. Microflora can both be regulated by immune mechanisms and the human genome (Levy et al., 2015), as well as be determined by environmental conditions including nutrition and other host factors (Hanski et al., 2012).

The human body's symbiotic relationship with microflora, which includes a range of bacteria, viruses, protozoa, and fungi that colonize all of the vital organs of the body, plays pivotal roles in human health (Cote et al., 2015) including a relationship with metabolism and the cause of human diseases. Recent studies reveal the role of gut microflora in metabolic diseases and tumors, and an increasing number of studies have been conducted to link the role of gut microflora to neurological disorders (De Vos and De Vos, 2012; Kim et al., 2013). Furthermore, the evidence is steadily increasing concerning the presence of an intense bidirectional interaction between intestinal microbes and the nervous system, their impact on brain activity and behavior, and the levels of neurotransmitter receptors and nerve agents (Bercik et al., 2011; Cryan and Dinan, 2012).

Parkinson's disease (PD) is one of the most prevalent degenerative neurological illnesses. Little is known about its causes, and the effect of environmental factors on PD genetic predisposition is not clear (Scheperjans et al., 2015). Recent studies demonstrate changes in the gut microflora of patients with PD that can serve as a biomarker of PD or its potential triggers that lead to α-synuclein dysfunction (a component of Lewy bodies), which is related to neurodegeneration in PD (Keshavarzian et al., 2015; Scheperjans et al., 2015). Furthermore, a fresh *in vivo* study also confirmed that gut microflora dysbacteriosis might related to rotenone-induced toxicity in the tested animals (Yang et al., 2017). Also for the human beings studies that done in western countries have presented that patients with PD show gut microflora dysbacteriosis (Keshavarzian et al., 2015; Scheperjans et al., 2015; Heintz-Buschart et al., 2017; Hill-Burns et al., 2017; Hopfner et al., 2017; Petrov et al., 2017). The aim of this study was to compare the structure of the gut microflora in PD patients and healthy controls at different stages using next-generation DNA sequencing tools, as well as to explore links between PD clinical features and antiparkinsonian medications on gut microflora.

## Materials and Methods

PD subjects (*n* = 72) were recruited from the China-Japan Friendship Hospital (Beijing), and either the spouses or family members of the subjects were considered healthy controls (*n* = 68) for the study (termed HCs). PD patients were divided into two subgroups: 59 patients suffered PD for ≥1 year (termed OPD), and 13 were new PD patients (termed NPD). The fecal samples of NPD patients were collected before treatment and 3, 5, 7, and 14 days after treatment. All subjects provided written informed consent for the use of their fecal samples for research. PD was diagnosed by experienced neurologists according to the UK Brain Bank Criteria (Hughes et al., 1992). The study protocol was approved according to the declaration of Helsinki and was achieved by the Ethics Committee of the Institute of Microbiology, Chinese Academy of Sciences.

HCs matched the PD groups by age and lifestyle. Standard mentality screening tests (including a structured caregiver interview and physical examination, a medical history, neuropsychological testing, and brain MRI) were performed for all PD patients (Egshatyan et al., 2016). The tests for parkinsonian symptoms were scored using the Unifed Parkinson's Disease Rating Scale (UPDRS) (Robichaud et al., 2009) and the Hoehn and Yahr scale (H-Y) (Goetz et al., 2004; Robichaud et al., 2009). Non-motor symptoms were evaluated using the Non-Motor Symptoms Scale (NMS) (Chaudhuri et al., 2007). The Wexner Constipation Scoring System was used to qualify constipation severity (Agachan et al., 1996). Anxiety and depression signs were scored via the Hamilton Depression Scale (HAMD) (Beneke, 1987). All clinical examinations were approved by the consensus of a multidisciplinary team. Controls underwent the same diagnostic procedures. Rejection conditions for all subjects included any gastrointestinal disease or neurodegenerative diseases; unstable medical, neurological, or psychiatric illness; and the use of any antibiotics or probiotics within 90 days before collection of the samples (Keshavarzian et al., 2015). Treatment data were recorded from the medical reports by the treating neurologists and involved only the medications that the patient was recommended for the treatment of PD at the time of this study.

## Results

### Subjects

The characteristics of the contributors are presented in Table 1. There were no significant differences between the PD and HC groups in terms of body mass index (BMI), gender, or age. No study subject had an infectious disease or required a special diet. Additionally, dietary data collected for all subjects showed no significant differences in macronutrients, dietary fiber, or total calorie intake between both groups.

**Table 1:** Demographic characteristics of the participants.

	**Healthy control**	**Old PD patients**	**New PD patients**	**Total PD *P*-value**
*n*	68	59	13	
Gender (Male/Female)	32/36	29/30	7/6	
Age (mean ±SD)	64.3 ± 4.8	65.1 ± 4.1	64.1 ± 3.5	0.89
Body -mass -index (kg/m^2^, median)	24.7	24.5	24.2	0.74
Constipation (Wexner score)	2	7	3	<0.05
Medication				
Levodopa	–	100%	100%	
Warfarin	10.2%	6%	15.4%	
Dopamine agonists	–	37.3%	–	
MAO-B inhibitor	–	22%	–	
Amantadine	–	18.6%	–	
COMT inhibitor	–	8.4%	–	
**Severity**				
UPDRS		Normal: 22 Moderate: 15 Severe: 22	Normal: 5 Moderate: 4 Severe: 4	<0.001
H-Y		Normal: 14 Moderate: 24 Severe: 21	Normal: 5 Moderate: 4 Severe: 4	<0.001
NMS		Normal: 25 Moderate: 17 Severe: 17	Normal: 5 Moderate: 3 Severe: 5	<0.001
HAMD		Normal: 28 Moderate: 20 Severe: 11	Normal: 4 Moderate: 4 Severe: 5	<0.001

*SD, standard deviation; UPDRS, Unifed Parkinson's Disease Rating Scale; H-Y, Hoehn and Yahr scale; NMS, Non-Motor Symptoms Scale; HAMD, Hamilton Depression Scale*.

### Alpha and Beta Diversity Between the PD and HC Groups

**Figure 1:**
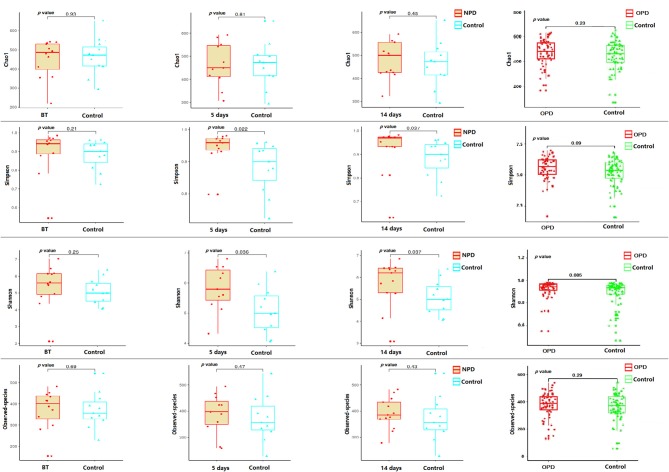
α-diversity indices of the fecal microbiome in the PD and their corresponding HC groups. Box plots depicting differences in the fecal microbiome diversity indices between the NPD, OPD, and HC groups according to the Chao1 index, observed species index, Shannon index, and Simpson index based on the OTU counts. No statistically significant differences were found with respect to commonly used α-diversity indices between the NPD, OPD, and HC groups. Each box plot represents the median, interquartile range, minimum, and maximum values. BT, before treatment. *P* level is corrected for multiple comparisons.

Taxonomic pattern evaluation of the gut microflora in PD patients (Figure 1) revealed that the level of α-diversity indices (Table S1) was significantly higher in the NPD group than those of the HC group using the Shannon (*P* = 0.038) and Simpson (*P* = 0.031) indices (Figure 1). We analyzed the taxonomic composition of the metagenomic populations of the gut microflora samples from patients with PD compared to those from the HC group using Principal Coordinate Analysis (PCoA). Significant differences were found in β-diversity based on the unweighted (qualitative, *P* = 0.003) but not the weighted (quantitative, *P* = 0.44) UniFrac between the OPD and HC groups (Figure S1), which indicates that the fecal microbial structure in the OPD group was significantly different than that of the HC group in condition of the presence of OTU. On the other hand, significant differences were found in β-diversity based on the weighted but not the unweighted UniFrac between the NPD and HC groups (Figure S2), which indicates that the fecal microbial structure in the NPD group after treatment was significantly different from that of HCs. Thus, the fecal microbial structure of the NPD group became significantly different from that of HC group in terms of OTU abundance after treatment. Reduction in taxonomic diversity of gut microflora in patients with PD can be a consequence of latent inflammatory processes in the digestive tract, which is reflected by several investigators as a trigger factor for α-synuclein misfolding in gut neurons (Keshavarzian et al., 2015; Scheperjans et al., 2015).

### Family- and Genus-Level Comparisons of the PD Groups and HCs

At the family level, comparing the taxonomic composition of the gut microflora revealed significant differences. For instance, the gut microflora of OPD patients contained high levels of Porphyromonadaceae, Christensenellaceae, Clostridialesvadin BB60 group, and Rikenellaceae and less Prevotellaceae and Lactobacillaceae compared to the corresponding HCs. Furthermore, significantly high levels of Turicibacteraceae, Ruminococcaceae, and Rikenellaceae were found in the NPD group compared to the corresponding HCs (Figure S3).

**Figure 2:**
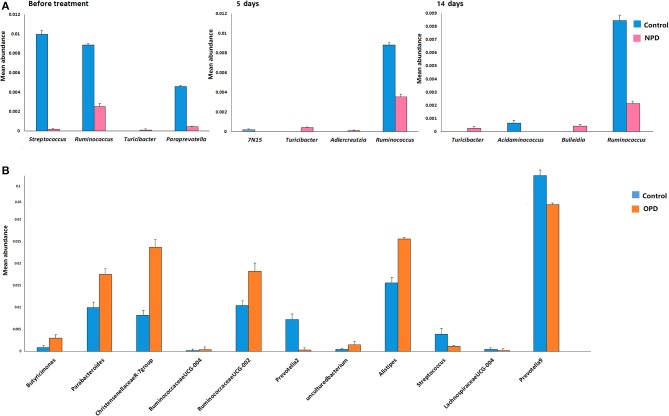
Genus-level comparison among groups of different disease stages. **(A)** Comparison of the mean abundance of the top dominant bacterial genera in the HC and NPD groups. **(B)** Control and OPD. Statistical analyses were performed by the Metastats method. Values are expressed as the mean ± SEM. Significance is considered at *P* < 0.05.

**Figure 3:**
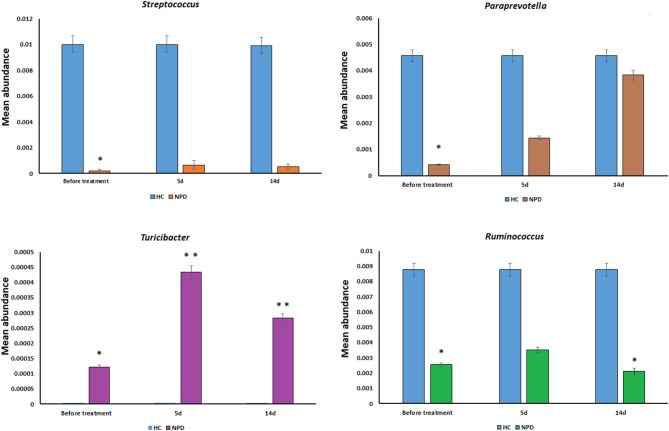
Genus-level comparison among NPD samples collected at different times. The changes in the mean abundance of the top dominant bacterial genera in the NPD group before and after treatment. Statistical analyses were performed by the Metastats method. Values are expressed as the mean ± SEM. *P < 0.05 and **P < 0.01.

Differences were also observed at the genus level. The microflora of patients with OPD was characterized by significantly higher levels of *Butyricimonas, Parabacteroides, Christensenellaceae R-7 group, Ruminococcaceae UCG*, and *Alistipes* compared to the corresponding HCs. Whereas, the HC group was characterized by a higher content of *Prevotella 2, Streptococcus, Lachnospiraceae UCG-004, and Prevotella 9* compared to the OPD group (Figure 2). Moreover, HCs had a slightly (but significantly) lower abundance of *Bulleidia, Turicibacter*, and *Adlercreutzia* and higher levels of *Streptococcus, Ruminococcus, Paraprevotella, 7N15*, and *Acidaminococcus* than the NPD group (Figures 2, 3).

### Taxa Variation Between the PD and HC Groups

Linear Discriminant Analysis (LDA) Effect Size (LEfSe) analysis was applied to the comparison of the microflora between the PD and HC groups. LEfSe is usually considered to recognize the effect size and occurrence of region-specific OTUs between dissimilar groups (Segata et al., 2011; Aboud et al., 2013). Our results suggested a remarkable difference in fecal microflora between the PD and HC groups, and we particularly focused on differences in the taxa at the genus level. The mean abundances of the genera *Lactobacillus, Streptococcus, Enterobacter, Citrobacter, Hungatella, Erysipelatoclostridium*, and *Veillonella* were higher in the HC group than in the OPD group, while a significantly higher abundance of the genera *Bifidobacterium, Alistipes, Klebsiella, Sellimonas, Catenisphaera*, and *Tyzzerella* was noted in the OPD patients compared to HCs [LDA score (log_10_) > 2, Figure S4I]. Concerning NPD duration, different relative abundances were observed before and after treatment, as well as during the time of treatment. Our explorative analysis suggested that the family Streptococcaceae and genera *Streptococcus* and *Paraprevotella* were more abundant in HCs than in NPD patients before treatment (Figure S4IIA). Ruminococcaceae where more abundant in NPD patients than their corresponding HCs at 5 days (Figure S4IIB) and 14 days (Figure S4IIC) after treatment.

### Clinical Correlations With the PD Microflora

**Figure 4:**
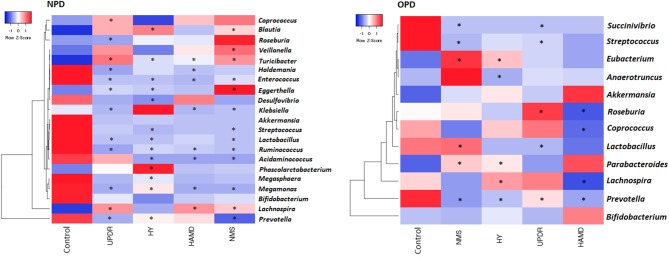
Heatmaps showing correlations between microflora genera and PD clinical characteristics. Heatmaps based on the abundance (sequence counts) of the microflora genera show the correlations between the fecal microflora and NPD\OPD group clinical characteristics, particularly severity and non-motor symptoms. PD severity includes H-Y stage and UPDRS part III scores. Non-motor symptoms of PD are represented by NMS scores and depression (HAMD scores). The intensity of the color represents the correlation with the corresponding HC groups (negative score, blue; positive score, red). H-Y stage, Hoehn, and Yahr stage; UPDRS, Unified Parkinson's Disease Rating Scale; NMS, Non-motor symptoms; HAMD, Hamilton Depression Scale. UPDRS scores were obtained during the on-phase at the outpatient clinic. Spearman test, *P ≤ 0.05.

We correlated the composition of the gut microflora with PD duration, disease severity, constipation, BMI, and age. The severity of PD was divided into three groups: normal (including normal and mild), moderate, and severe symptoms. The genus *Eubacterium* was positively correlated to severity, whereas the genera *Prevotella* and *Lachnospira* displayed a negative correlation (Figure 4).

We observed a slight but significant negative correlation between NPD severity and different genera (Figure 4). Of the 130 correlations between taxa, age, and BMI, we found only 10 significant correlations: age correlated with two taxa in PD and none in HC subjects, and BMI correlated with four taxa in PD and one in HC subjects (data not shown).

### Predictive Functional Analysis

**Figure 5:**
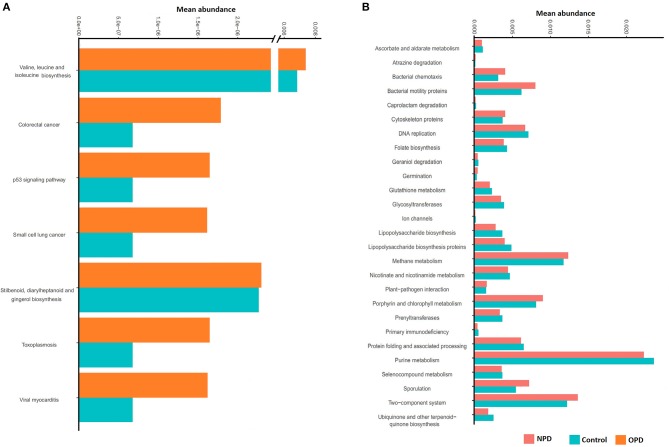
Functional predictions for the fecal microbiome of the PD and HC groups. In STAMP, differences in abundances between the OPD **(A)**, NPD **(B)**, and their corresponding HCs were compared using White's non-parametric *t*-test. Confidence intervals were estimated using a percentile bootstrapping method.

PICRUst based on closed-reference OTU was applied to predict the abundances of functional categories KEGG orthologs (KOs). Several KOs were identified with significantly different abundances in the fecal microbiome between the NPD group during treatment and HCs (*P* < 0.05; Figure S5). At the level of KEGG pathways, the function of microbial genes, including pathways involved in Colorectal cancer, Small cell lung cancer, Stilbenoid, diarylheptanoid and gingerol biosynthesis, Toxoplasmosis, Valine, leucine and isoleucine biosynthesis, Viral myocarditis, and the p53 signaling pathway were significantly increased (*P* < 0.05) in the OPD group relative to the HC group (Figure 5A). In addition, the microbial gene functions, including pathways involved in atrazine degradation, bacterial chemotaxis, bacterial motility proteins, cytoskeleton proteins, germination, methane metabolism, plant-pathogen interaction, porphyrin and chlorophyll metabolism, sporulation, and two-component system were significantly higher in the NPD group (*P* < 0.05) relative to the HC group, whereas caprolactam degradation, ascorbate and aldarate metabolism, DNA replication, folate biosynthesis, geraniol degradation, glutathione metabolism, glycosyltransferases, ion channels, lipopolysaccharide biosynthesis, lipopolysaccharide biosynthesis proteins, nicotinate and nicotinamide metabolism, prenyltransferases, primary immunodeficiency, protein folding and associated processing, purine metabolism, seleno-compound metabolism, and ubiquinone and other terpenoid-quinone biosynthesis pathways were significantly higher (*P* < 0.05) in the HC group (Figure 5B).

## Discussion

Many studies indicate that syndromes linked to metabolic functions and PD share common characteristics, such as insulin resistance and chronic inflammation associated with similar impairments in brain function and anatomy (Willette et al., 2015; Pistollato et al., 2016). Such studies also highlight the role of the gut-brain axis in regulating the immune response, host metabolism, and the functions of the nervous system. Under dysbacteriosis situations, intestinal flora become unhealthy. As a result, chronic inflammation begins, along with a large range of immune reactions and metabolic issues that may contribute to the beginning of metabolic syndrome, eczema, autism, obesity, Alzheimer's disease, and PD.

Healthy spouses of the PD patients were included as controls in our study to minimize the confounding effects of dietary differences on the microflora. Each spouse involved in our study lived in the same household as the PD patient for decades. The spouses shared more similar communities of microflora than separated individuals living in different households (Song et al., 2013), suggesting that a shared environment or diet affects the similarity of the fecal microflora (Yatsunenko et al., 2012).

We found that PD patients had a distinct fecal microbiome from their healthy spouses, and this may indicate a link between the gut microflora dysbacteriosis linked to PD (Qian et al., 2018). In our results, α-diversity indices of the fecal microflora in OPD patients displayed no statistically significant differences compared to previous studies investigating fecal microflora in PD patients using 16S-based techniques [i.e., with the α-diversity indices in Finnish (Scheperjans et al., 2015) and Germany populations (Hopfner et al., 2017)], which is consistent with our results. However, α-diversity indices were significantly higher in NPD patients, demonstrating that the diversity and richness of the gut microflora in NPD patients was significantly higher than that in the HC group. This result is in consistent with another Chinese PD study (Qian et al., 2018). Additionally, Keshavarzian et al. report that fecal sample α- diversity indices are greater in American PD patients than in controls (Keshavarzian et al., 2015).

Furthermore, we confirmed the significant differences in β-diversity indices that are reported in other studies (Qian et al., 2018). β-diversity indices offer powerful proofs that the gut microflora in OPDs is altered from that of HCs. Significant differences in bacterial composition were revealed after comparison of fecal samples from PD patients and corresponding HCs, which agrees with earlier reported data in a Finnish population (Keshavarzian et al., 2015). Interestingly, the distance between dots on the plot (indicating the degree of similarity of taxonomic composition) of the NPD samples was altered for most subjects during the time of the study, meaning that the similarity to the HC group was altered after treatment with levodopa. This indication for an interaction between PD medications and the microflora is not surprising, considering the growing evidence of the role of the gut microflora in the metabolism and uptake of medical treatments, as well as the profound effects that medications can have on the structure of the microbiome. A prior study of NPD patients links antiparkinsonian drugs to changes in the abundance of some taxa, which may be related to the bidirectional interaction between the nervous system and microflora and the impact of brain activity, behavior, and medication on the gut and their microflora. The current conclusions lend support to the concept that the structure of the gut microflora may hold new information for assessing the activity and toxicity of PD medications. Further studies are required to evaluate the effects of levodopa and other PD treatments on a greater number of treated and untreated patients (Bercik et al., 2011; Cryan and Dinan, 2012).

Including more sequencing reads into the statistical analysis than earlier reports, our research is focused on differences at the family and genus levels. The ability to provide genus identification is one of the most attractive applications of 16S rRNA gene sequence informatics (Scheperjans et al., 2015). The microflora of patients with OPD was confirmed by many of the reported links containing increased levels of *Alistipes* and *Bifidobacterium* and reduced levels of *Lactobacillus, Prevotella*, and *Lachnospiraceae UCG* (Scheperjans et al., 2015; Bedarf et al., 2017; Hill-Burns et al., 2017). *Prevotella* and *Lactobacillus* are gradually gaining attention, especially due to their capability to degrade plant polysaccharides, affect glucose intolerance, decrease short chain fatty acids (SCFAs), and links to osteomyelitis and metabolic endotoxemia (Scher et al., 2013; Zmora et al., 2019). The NPD group presented higher levels of *Bulleidia, Turicibacter*, and *Adlercreutzia* and lower levels of *Streptococcus, Ruminococcus*, and *Paraprevotella* compared to the HCs. These differences between the NPD and OPD groups may be due to changes in lifestyle and the effect of PD medication during the course of the study. Nevertheless, we could not elucidate the detailed roles of the gut microflora in the pathogenesis of PD from this cross-sectional study. Recently, an animal study from another Chinese team discovered that gut microflora dysbacteriosis has impacts even earlier than α-synuclein formation in the brains of mice, which may hint at the role of gut microflora in the pathogenesis of PD (Yang et al., 2017).

Nevertheless, different studies worldwide that focus on the link between gut microflora and PD report different results, and thus, proposing a comprehensive conclusion is difficult. Several aspects, such as different 16S primer sets and sequencing techniques, may be responsible for the differences in the reported abundances of organisms in the microflora (Hill-Burns et al., 2017; Qian et al., 2018). In addition, the structure of the gut microflora is hypothesized to dramatically differ among populations and individuals, consistent with ethnic origins, geography, host genetics, age, and other factors (Escobar et al., 2014). The recruitment of healthy spouses as controls is another key advantage that may largely influence our results.

A number of reports link the reduction of SCFAs to the pathogenesis PD (Hill-Burns et al., 2017). SCFAs are produced by microbes in the gut, notably Lachnospiraceae. We found decreased levels of Lachnospiraceae in PD, which is compatible with the depletion of SCFAs. Our data indicated that some microbes were associated with clinical features of PD, i.e., *Eggerthella, Prevotella, Turicibacter, Lactobacillus*, and *Enterococcus* were correlated with disease severity, medication, and non-motor symptoms. This varies from the results of Keshavarzian's study, which shows that no genus in the feces is associated with H-Y stages, UPDRS total, and part III scores (Keshavarzian et al., 2015). Moreover, we found that the genera *Streptococcus* and *Prevotella* were negatively associated with medical treatment, whereas *Turicibacter* was positively correlated, indicating that the microflora might influence drug metabolism or that drugs might affect the microflora (Hill-Burns et al., 2017).

## Conclusions

Our finding that some fecal microflora were closely related to PD clinical characteristics may enhance our comprehension of PD pathogenesis. Well-designed human studies can show how PD treatments change the microflora and the side effects that may follow. Such studies can also demonstrate how the structure of the microflora could affect metabolism and, later, the toxicity and efficacy of different treatments.

## Data Availability Statement

The datasets generated for this study can be found in the NCIB Repository: https://www.ncbi.nlm.nih.gov/biosample/. Accession numbers: 13258423–13258555.

## Ethics Statement

The sampling was performed in accordance with Chinese laws.

## Author Contributions

RA and BZ developed the study concept and design, performed the data analysis. RA, JL, HT, LW, KW, and MJ collected the samples. RA and NL completed the DNA preparation and environmental genomic experiments. RA, SL, and FL analyzed the data. All authors drafted the manuscript. All authors read and approved the final manuscript and agree to be accountable for all aspects of the work in ensuring that questions related to the accuracy or integrity of any part of the work are appropriately investigated and resolved.

### Conflict of Interest

The authors declare that the research was conducted in the absence of any commercial or financial relationships that could be construed as a potential conflict of interest.
