# Supplementary material for: Analysis of the Gut Microflora in Patients With Parkinson's Disease
Source: Front Neurosci. 2019 Nov 22;13:1184. doi: 10.3389/fnins.2019.01184 (PMC6883725; doi:10.3389/fnins.2019.01184)
Supplement: Supplementary file 1 [file Data_Sheet_1.PDF]

Analysis of the gut microflora in patients with Parkinson's disease

Rashad Alkasir[1,†]; Miao Jin[4,†]; Fei Liu[1]; Na Lv[1]; Jing Li[1]; Hua Tao[1]; Kang Wang[4]; Li Wang [4]; Lu Wang[4]; Jun Li [4]; Ying Hao[4]; Shihao Liang[1]; Baoli Zhu[1,2,3]*

Supplementary Materials and Methods

Collection of samples and extraction of DNA

The subjects collected the fecal samples at home or at hospital into collection tubes pre-filled with Stool DNA Stabilizer (Tiangen Biotech Co. Ltd., Beijing). Within three days, the tubes were transferred to the laboratory and stored at -80C until further processing. TIANamp stool DNA kit (Tiangen Biotech Co. Ltd., Beijing) was used to extract the total microbial DNA basing on the manufacturer's guidelines as described previously (Egshatyan et al., 2016). DNA samples were quantifed using a Qubit2.0 Fluorometer (Invitrogen,USA) and DNA quality was confirmed using 0.8%agarose gel electrophoresis.

Amplification and sequencing of 16S rRNA gene

Isolated fecal DNA was used as a template for the amplification of theV3–V4 region of 16S ribosomal RNA (rRNA) gene. As previously discriped (Takahashi et al., 2014), theV3andV4 regions were amplified using forward primers containing the sequence 5′-CCTACGGGNBGCASCAG-3′ and reverse primers containing the sequence 5′-GACTACNVGGGTATCTAATCC-3′. Besides the 16S target-specific sequence, primers also contained adaptor sequences allowing uniform amplification of a highly complex library ready for downstream NGS sequencing on Illumina Hiseq2500 (Li et al., 2017). Illumina MiSeq instrument was used to multiplex and load DNA libraries basing on manufacturer's guidelines (Illumina,

USA). Sequencing was performed using a 2×250 (PE) configuration; image analysis and base calling were conducted with Hiseq2500 Control Software on the Hiseq instrument.

Bioinformatics and statistical data analyses

FLASH (Fast Length Adjustment of SHort reads) was used to merge paired-end reads from next-generation sequencing (Magoc and Salzberg, 2011). Low quality reads were filtered by fastq_quality_filter (-p 90 -q 25 -Q33) in FASTX Toolkit 0.0.14 and chimera reads were removed by USEARCH 64 bit v8.0.1517. The number of reads for each sample was normalized based on the smallest size of samples by random subtraction. Operational taxonomic units (OTUs) were aligned by UCLUST algorithm with a 97% identity and taxonomically classified using the SILVA 16S rRNA database v128. Alpha and beta diversities were generated in the Quantitative Insights Into Microbial Ecology (QIIME) and calculated based on weighted and unweighted Unifrac distance matrices.

The Metastats method was used to evaluate abundance features between PD and control groups (http://metastats.cbcb.umd.edu/detection.html). The R package “heatmap” and “stats” programs were used to generate of the heat maps and hierarchical clustering respectively (Li et al., 2017).

Quality filtering of the reads and their taxonomic classification were accomplished using QIIME software (Caporaso et al., 2010) by comparing the obtained reads to the HITdb database (Ritari et al., 2015). QIIME software was used to evaluate Alpha- and beta-diversities. Rarefaction of the samples was performed at a depth of 20000 sequences per sample with further calculation of the chao1 index in the PD and control groups to evaluate alpha diversity of microbial populations. Beta-diversity among populations was evaluated with principal coordinates analysis (PCoA) on a

weighted Unifirac metric. ANalysis Of SIMilarity (ANOSIM) was applied to measure the *P* value and effect size *R*, evaluating statistical significance with 19999 permutations.

Functional analysis of predicted metagenomes

We used Phylogenetic Investigation of Communities by Reconstruction of Unobserved States (PICRUSt) (Langille et al., 2013) to infer metagenome composition in the samples, following the recommended pipeline of normalizing OTUs by copy number (to account for differences in number of copies of 16S rRNA between taxa), predicting functions using Kyoto Encyclopedia of Genes and Genomes (KEGG) (Kanehisa et al., 2004) orthologs, and grouping predicted pathways by KEGG hierarchical level.

Statistical analysis

Statistical analysis of bacterial taxa was achieved by a non-parametric White's *t* test using STAMP software (Whitley and Ball, 2002). Multiple comparisons adjustment of the *P* values was performed using the Benjamini—Hochberg FDR-controlling procedure. Differences were considered statistically significant at $p \leq 0.05$ at 20,000 permutations.

References

caporaso, J. G., Kuczynski, J., Stombaugh, J., Bittinger, K., Bushman, F. D., et al. (2010). QIIME allows analysis of high-throughput community sequencing data. *Nat Methods,* 7**,** 335-6.

Egshatyan, L., Kashtanova, D., Popenko, A., Tkacheva, O., Tyakht, A., et al. (2016). Gut microbiota and diet in patients with different glucose tolerance. *Endocr Connect,* 5**,** 1-9.

Kanehisa, M., Goto, S., Kawashima, S., Okuno, Y. & Hattori, M. (2004). The KEGG resource for deciphering the genome. *Nucl acids res,* 32**,** D277-D280.

Langille, M. G., Zaneveld, J., Caporaso, J. G., Mcdonald, D., Knights, D., et al. (2013). Predictive functional profiling of microbial communities using 16S rRNA marker gene sequences. *Nat Biotechnol,* 31**,** 814-21.

Li, W., Wu, X., Hu, X., Wang, T., Liang, S., Duan, Y., Jin, F. & Qin, B. (2017). Structural changes of gut microbiota in Parkinson's disease and its correlation with clinical features. *Science China Life Sciences,* 60**,** 1223-1233.

Magoc, T. & Salzberg, S. L. (2011). Flash: fast length adjustment of short reads to improve genome assemblies. *Bioinformatics,* 27**,** 2957-63.

Ritari, J., Salojarvi, J., Lahti, L. & De Vos, W. M. (2015). Improved taxonomic assignment of human intestinal 16S rRNA sequences by a dedicated reference database. *BMC Genomics,* 16**,** 1056.
Takahashi, S., Tomita, J., Nishioka, K., Hisada, T. & Nishijima, M. (2014). Development of a Prokaryotic Universal Primer for Simultaneous Analysis of Bacteria and Archaea Using Next-Generation Sequencing. *PLoS ONE,* 9**,** e105592.
Whitley, E. & Ball, J. (2002). Statistics review 6: Nonparametric methods. *Critical Care,* 6**,** 509-513.

Supplementary figures

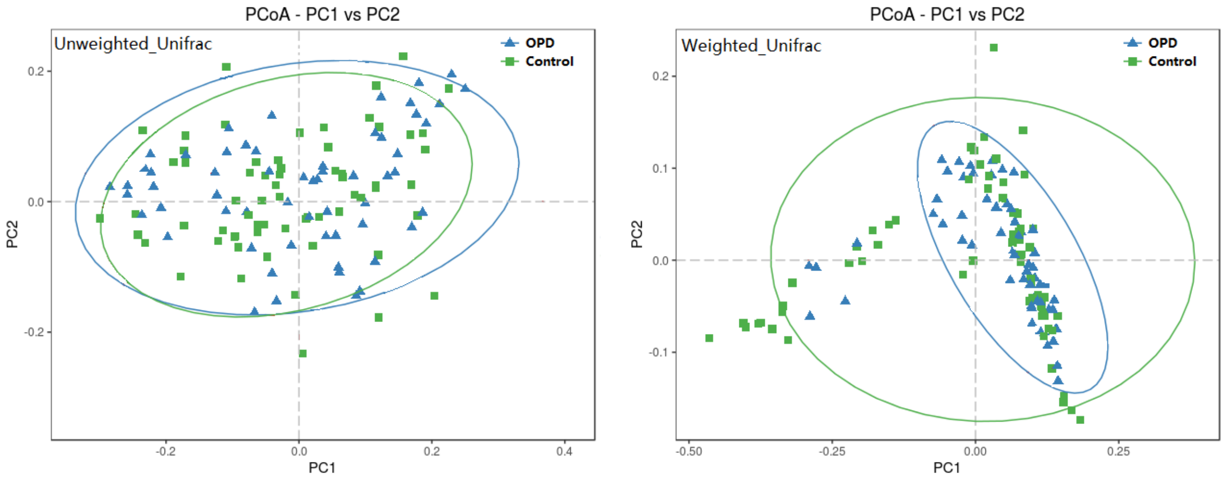

FIGURE S1. **Unweighted and weighted ANOSIMs and PCOA based on the distance matrix of UniFrac dissimilarity of the fecal microbial communities in the OPD and healthy groups.** Respective ANOSIM *R values* show the community variation between the compared groups, and *P values* are indicated. The axes represent the two dimensions explaining the greatest proportion of variance in the communities. Each symbol represents a sample. The distance between dots on the plot indicates the degree of similarity of taxonomic composition of the samples. OTU, operational taxonomic unit; ANOSIM, analyses of similarities. *P* level is corrected for multiple comparison.

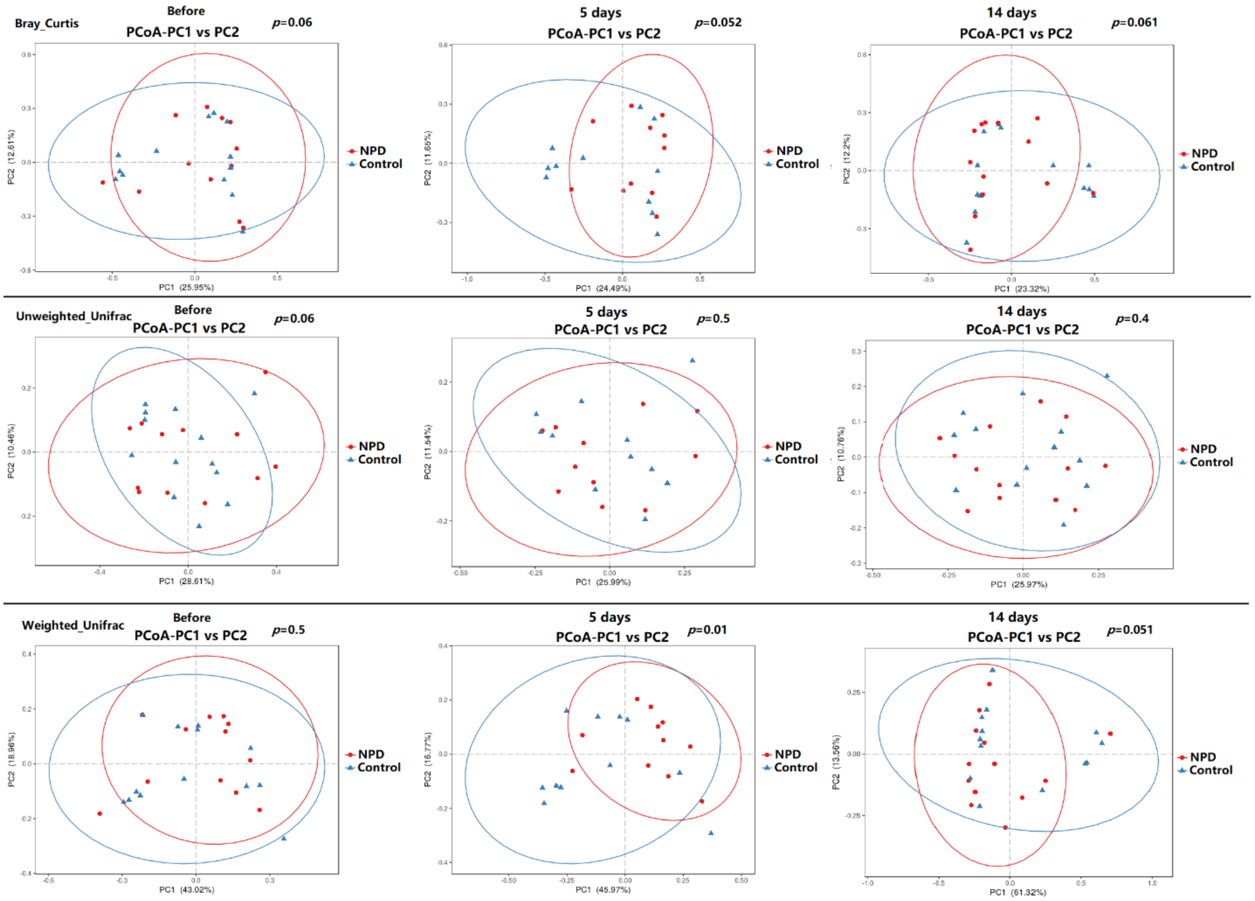

FIGURE S2. PCOA based on the distance matrix of UniFrac dissimilarity of the fecal microbial communities in the NPD and healthy groups. The axes represent the two dimensions explaining the greatest proportion of variance in the communities. Each symbol represents a sample. The distance between dots on the plot indicates the degree of similarity of taxonomic

composition of the samples. OTU, operational taxonomic unit; ANOSIM, analyses of similarities. *P* level is corrected for multiple comparison.

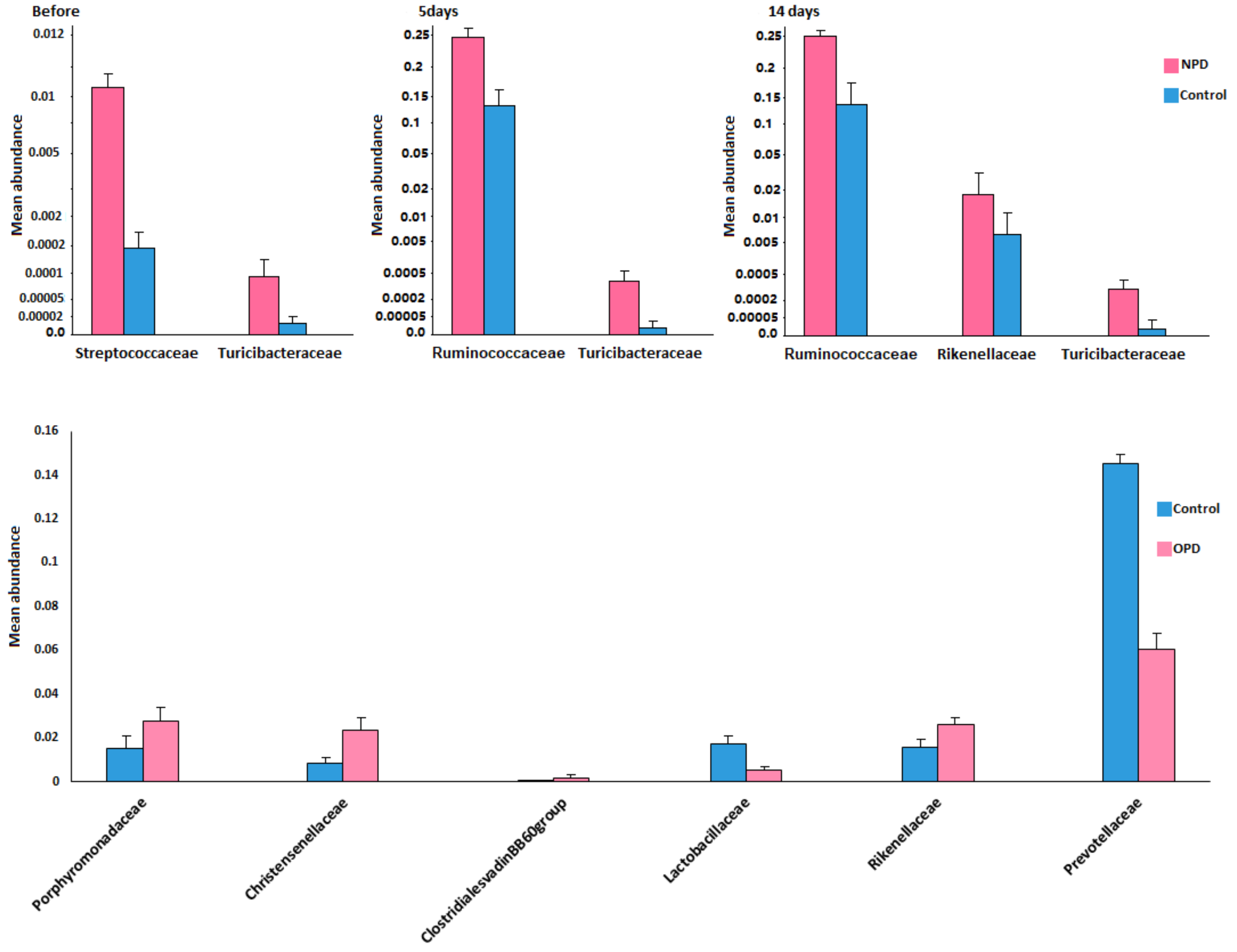

FIGURE S3. **Family level comparison among groups of different disease stages.** Comparison of the relative abundance of the top dominant bacterial families in groups of different disease stages. Statistical analysis was performed by Metastats method. Values are expressed as mean ± SEM. Significance is considered at $P < 0.05$.

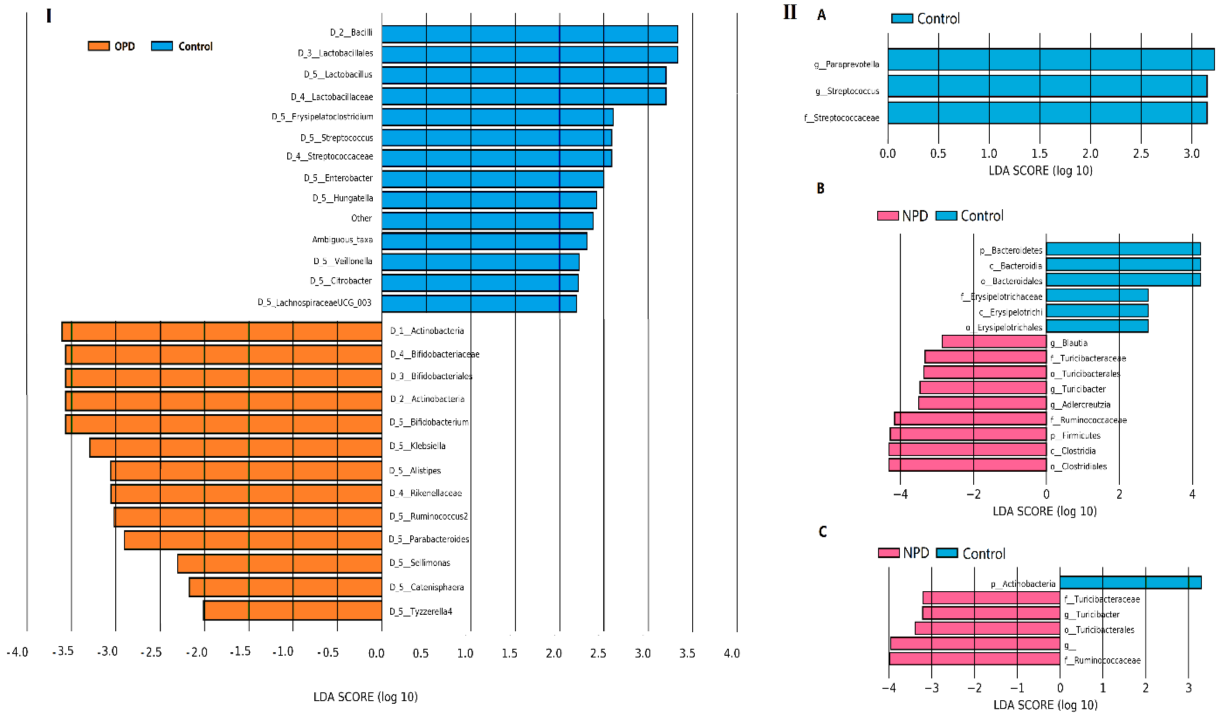

FIGURE S4. Taxonomic differences of fecal microbiota in OPD (I), NPD (II) and their healthy groups. Linear discriminant analysis (LDA) effect size (LEfSe) analysis revealed significant bacterial differences in fecal microbiota between the PD, and healthy groups at different levels. The LDA scores (log10) > 2 and $P < 0.05$ are listed. The explorative analysis suggested that two families were more abundant in OPD patients than in corresponded controls.

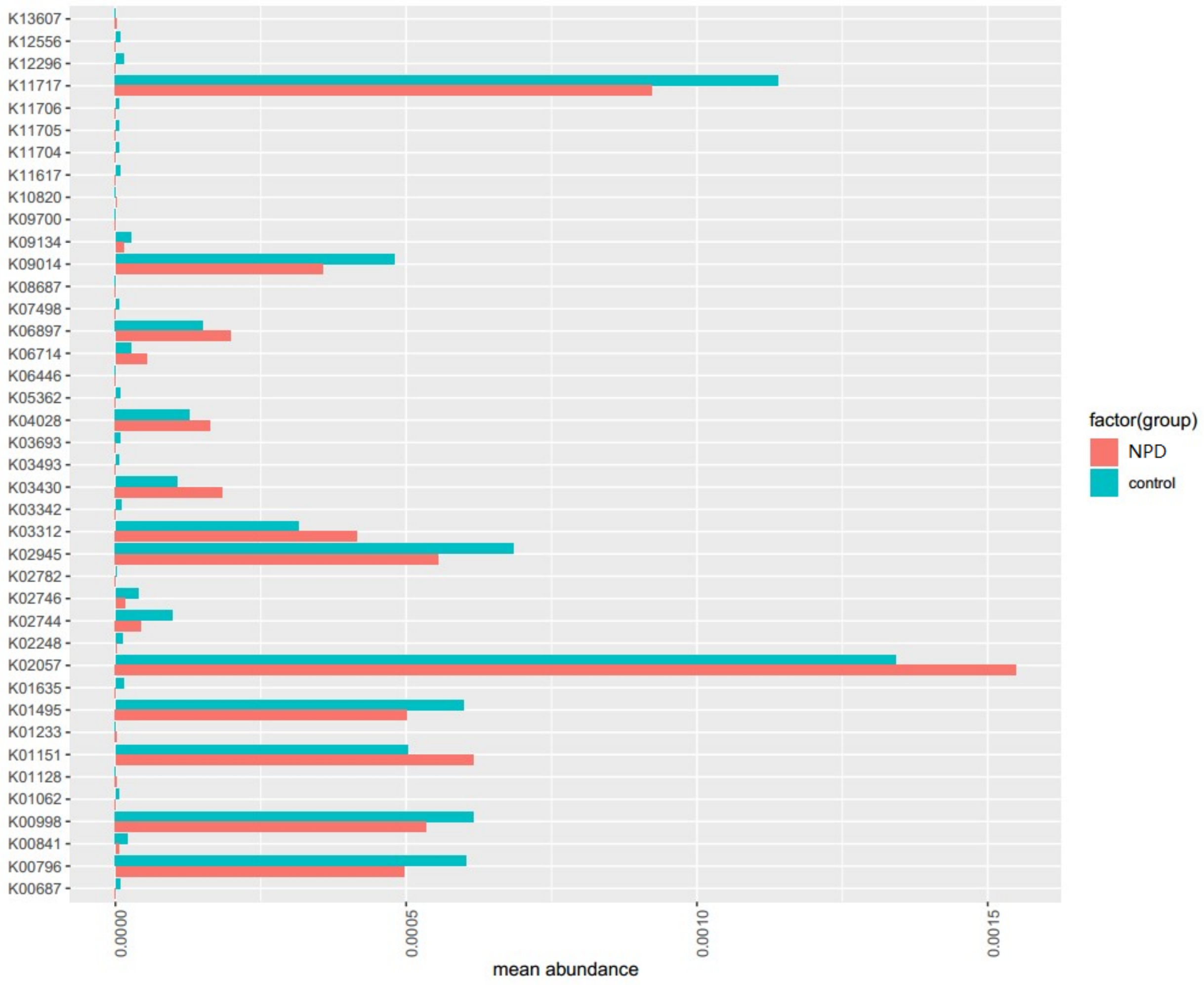

FIGURE S5. **Functional predictions for the fecal microbiome of the NPD before treatment and healthy groups.** The KOs with significantly different abundances in the fecal microbiome identified using the software package PICRUSt are shown ($P < 0.05$).

Supplementary tables

TABLE S1| Summary of sequencing data

	Health control	NPD patients	OPD patients
**Sequences(mean±SD)**	117778±43738.3	110715±46187.2	118033±39383.5
**OTUs(mean±SD)**	15588.91±1688.96	16146.8±1635.86	15252.85±1590.56
**Chao(mean±SD)**	452.17±111.92	475.92±81.66	461.31±91.75
**Shannon(mean±SD)**	5.24±1.04	5.71±0.92	5.51±0.71
**Good's coverage**	99.42%	99.44%	99.44%
